# LC-MS-Based Targeted Metabolomics for FACS-Purified Rare Cells

**DOI:** 10.1021/acs.analchem.2c04396

**Published:** 2023-02-22

**Authors:** Katharina Schönberger, Michael Mitterer, Katharina Glaser, Manuel Stecher, Sebastian Hobitz, Dominik Schain-Zota, Konrad Schuldes, Tim Lämmermann, Angelika S. Rambold, Nina Cabezas-Wallscheid, Joerg M. Buescher

**Affiliations:** † Max Planck Institute of Immunobiology and Epigenetics, Stübeweg 51, 79108 Freiburg, Germany; ‡ International Max Planck Research School for Immunobiology, Epigenetics and Metabolism (IMPRS-IEM), 79108 Freiburg, Germany; § Faculty of Biology, University of Freiburg, 79085 Freiburg, Germany; ∥ International Max Planck Research School for Immunobiology, Epigenetics and Metabolism (IMPRS-MCB), 79108 Freiburg, Germany

## Abstract

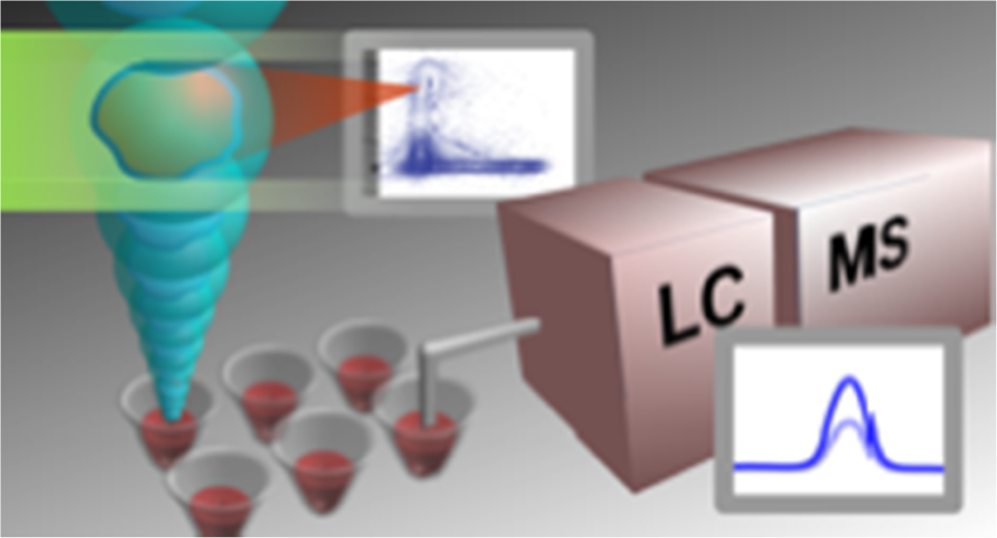

Metabolism plays a fundamental role in regulating cellular functions and fate decisions. Liquid chromatography-mass spectrometry (LC-MS)-based targeted metabolomic approaches provide high-resolution insights into the metabolic state of a cell. However, the typical sample size is in the order of 10^5^–10^7^ cells and thus not compatible with rare cell populations, especially in the case of a prior flow cytometry-based purification step. Here, we present a comprehensively optimized protocol for targeted metabolomics on rare cell types, such as hematopoietic stem cells and mast cells. Only 5000 cells per sample are required to detect up to 80 metabolites above background. The use of regular-flow liquid chromatography allows for robust data acquisition, and the omission of drying or chemical derivatization avoids potential sources of error. Cell-type-specific differences are preserved while the addition of internal standards, generation of relevant background control samples, and targeted metabolite with quantifiers and qualifiers ensure high data quality. This protocol could help numerous studies to gain thorough insights into cellular metabolic profiles and simultaneously reduce the number of laboratory animals and the time-consuming and costly experiments associated with rare cell-type purification.

The relevance of intracellular metabolism beyond its classical housekeeping function is increasingly recognized across many disciplines of the life sciences. Therefore, investigating metabolic aspects has gained increasing interest. Some recent reviews provide great examples from diverse fields such as epigenetics,^1,2^ cancer metabolism,^3^ immunology,^4,5^ and plant physiology^6,7^ and highlight the importance of intra- and extracellular metabolite abundances.

A broad range of analytical tools has been developed to probe intracellular metabolism. Tools that are specifically suited for this task include tracing of nutrients labeled with stable isotopes,^8−10^ time-resolved measurement of extracellular parameters such as pH and oxygen tension,^11,12^ in particular in combination with well-characterized metabolic drugs,^13^ and metabolomics, which commonly includes the quantification of intracellular metabolites to provide snapshots of the metabolic state of cells. The latter is particularly versatile, as analytical methods for many classes of metabolites have been described in the literature.^14^ Mass spectrometry (MS) coupled to gas- or liquid chromatography dominates the metabolomics field, while nuclear magnetic resonance (NMR) can hold its ground for some applications.

Typical metabolomics protocols require 10^5^–10^7^ cultured cells, 5–50 mg of tissue, or 5–50 μL of body fluid per sample. This amount of input material can be challenging if working with primary cells isolated from small animals or precious human patient samples. Depending on the cell type and source, only a few thousand cells can be isolated.

Hematopoietic stem cells (HSCs) are an example of such a rare cell type that is of particular interest in the field of leukemia therapy. A deep understanding of the HSC metabolism will provide novel insights into the regulation of hematopoietic homeostasis and leukemic transformation and aid in improving clinical applications such as ex vivo expansion for transplantation purposes and therapeutic treatment schemes. Depending on the surface markers used for identification, between 1000 to 5000 HSCs can be isolated from a single mouse.^15−17^

Scientists interested in the metabolism of a specific primary cell type face the challenge of obtaining metabolomics data that is (i) informative for their scientific question and (ii) specific for the target cell type. In tissues, different cell types vary in their metabolic content but cannot be separated from each other as they are often closely intertwined.^18,19^ MS imaging offers the possibility for spatially resolved metabolite analysis. However, this technology requires costly specialized equipment, and machines with single-cell resolution are still in their infancy.^20^ Moreover, the omission of chromatographic separation hinders the separation of isomers, and the acquisition of only MS1 data reduces the confidence in the annotation of ions.^21−23^ Isolation of defined populations of primary cells from a mix of cells is usually performed by fluorescence-activated cell sorting (FACS). Many cells have to be removed from their physiological environment to generate a suspension of single cells. Sample preparation and sorting of rare cells can take minutes to hours, during which the cells are typically exposed to nutrient-free buffers such as phosphate-buffered saline (PBS). This procedure is known to cause cellular stress and alter the metabolome.^24−26^ Subsequent cultivation of primary cells in vitro can reduce this starvation phenotype; however, the chosen culture conditions and the duration of the cultivation can also alter the metabolome^27,28^ and change the differentiation status of primary cells.^29^ Since many research labs lack equipment for MS imaging, we here opted to pursue an approach in which FACS-separation of primary cells is directly followed by liquid chromatography-mass spectrometry (LC-MS) metabolite detection. Minimizing exposure to nonphysiological conditions helps in obtaining useful metabolomics data.

Previously, cells from over 100 animals had to be pooled per sample to achieve sufficient signal intensity for rare cell types.^30^ In more recent studies, cells from a single mouse or pools of cells from 2 to 10 mice (depending on target metabolites and cell type) were used to obtain enough cells per sample for metabolomics measurements.^31,32^ The combination of using acetonitrile for metabolite extraction and using hydrophilic interaction chromatography (HILIC) for metabolite separation allows for simplified sample preparation without sample drying and thus less risk of metabolite loss. This, in combination with metabolite detection using modern mass spectrometers, enabled the quantification of metabolite in as little as 10,000 cells. In an alternative approach using nanoflow LC-MS and chemical derivatization, the 80–106 amine- and/or phenol-containing compounds were detected in 100–10,000 cultured cancer cells, respectively.^33^

In this work, we describe an optimized workflow for performing liquid chromatography-mass spectrometry (LC-MS)-based metabolomics on FACS-sorted cell populations (Figure 1). Coordinated optimization of all steps from the mixed cell suspension to LC-MS analysis allowed us to measure intracellular metabolites in as little as 100 cells, including some high-turnover metabolites such as uridine triphosphate (UTP) and S-adenosyl-methionine (SAM). To enable a broader coverage of the metabolome, we have optimized the protocol for samples of 5000 cells. Sorting directly into extraction buffer minimized exposure to nonphysiological conditions and preserved cell-type-specific differences. The generation of relevant blank reference samples, the addition of internal standards to every sample, and targeted metabolite detection by LC-QQQ-MS ensured high data quality. We hope that this work will contribute to reducing the number of laboratory animals required for biomedical research and maximizing the information that can be obtained from patient samples.

**Figure 1:**
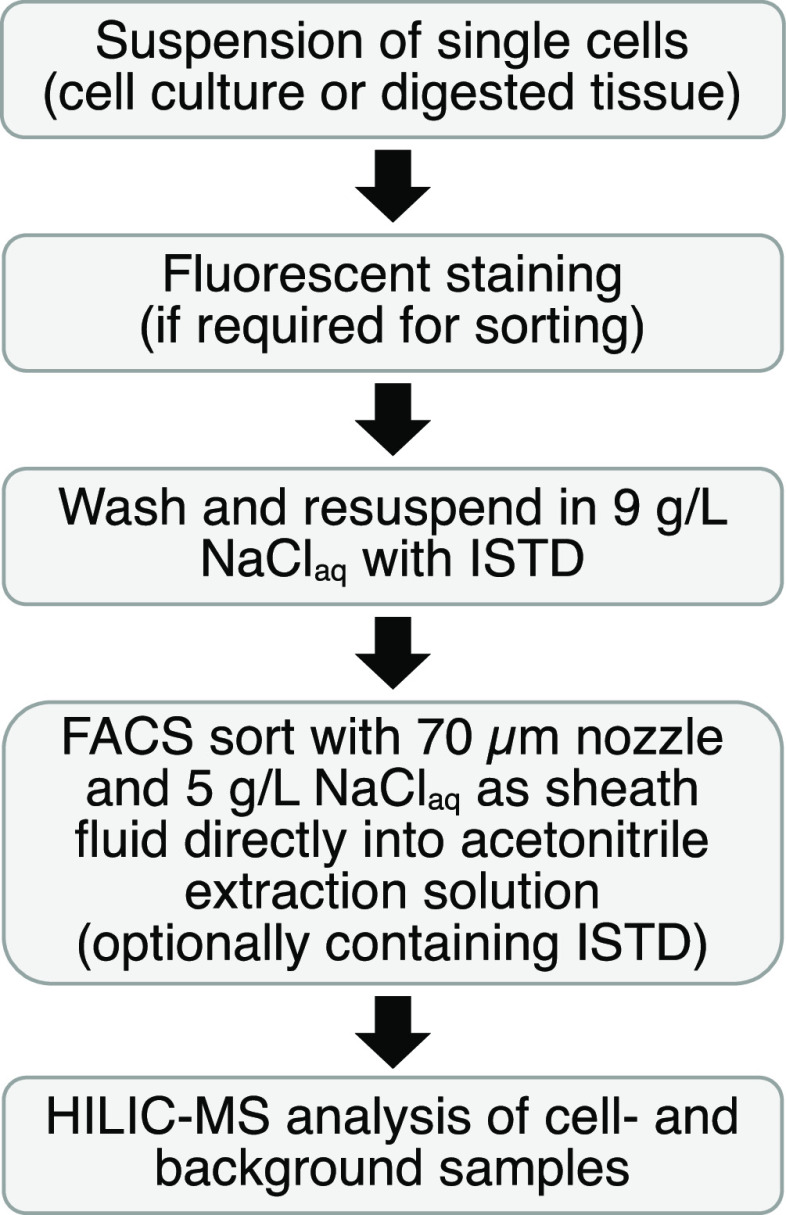
General workflow of the optimized protocol.

## Experimental Section

### Isolation of Murine Peripheral Blood Cells (T Cells and B Cells)

According to guidelines and animal protocols approved by the German authorities, mice were handled and bled from the cheek vein to withdraw 50 μL of peripheral blood. The blood was collected in an EDTA-coated 2 mL tube and kept on ice. Note that cells must be kept on ice from the moment of isolation.

Lysis of erythrocytes was performed by adding 300 μL of ice-cold ACK lysis buffer (Lonza, Cat# 10-548E) and incubating on ice for 10 min after transferring the cells into a conical FACS tube. The cells were then centrifuged at 400*g* for 5 min at 4 °C, and the supernatant was discarded. A second (and optional third) round of erylysis was performed until a defined pellet was visible. The reaction was stopped by adding 500 μL of ice-cold PBS (Sigma, Cat# D8537) to each sample. Following an additional centrifugation step at 400*g* for 5 min at 4 °C, and the supernatant was discarded.

### Isolation of Murine Bone Marrow Cells (Stem/Progenitor Cells)

Mice were handled and euthanized by cervical dislocation according to guidelines and animal protocols approved by the German authorities. Using forceps and scissors, the animal’s legs and spines were dissected. To isolate femurs, tibiae, iliae, and vertebrae, connective tissue was removed with a scalpel. The isolated bones were then kept on ice in PBS (Sigma, Cat# D8537)-filled 6-well plates until all bones were collected.

Note that cells must be kept on ice from the moment the bones are isolated from the mouse. Bones were gently crushed twice with 5 mL of ice-cold PBSs using a mortar and pestle until they turned completely white. The cell suspension was then filtered through a 40 μm sterile filter (Corning, Cat# 352340) into a 50 mL falcon tube and kept on ice.

Next, the lysis of erythrocytes was performed. Briefly, the cell suspension was centrifuged at 400*g* for 5 min at 4 °C, followed by the removal of the supernatant. The pellet was then resuspended into 2 mL of ice-cold ACK lysis buffer (Lonza, Cat# 10-548E) and incubated on ice for 5 min (incubation time may be increased up to 10 min depending on the size of the pellet). The reaction was then stopped by adding 10 mL of ice-cold PBS. Then, the cells were centrifuged at 400*g* for 5 min at 4 °C, and the supernatant was discarded.

Lineage negative (Lin−) cells were enriched using the Dynabeads Untouched Mouse CD4 Cells Kit (Invitrogen, Cat# 11415D). Briefly, cells were resuspended in a 500 μL of lineage cocktail (100 μL of the cocktail provided in the kit plus 400 μL of PBS) and transferred into a 2 mL tube. The incubation step was performed for 35 min on a rotating wheel at 4 °C. Meanwhile, 400 μL of Dynabeads were washed twice with 1 mL of PBS on a magnet and resuspended in 500 μL of PBS.

Next, the cells were washed with 12 mL of ice-cold PBS in a 15 mL falcon tube and centrifuged at 400*g* for 5 min at 4 °C. After the removal of the supernatant, cells were resuspended in 1 mL of ice-cold PBS and incubated together with the prepared Dynabeads for 20 min on a rotating wheel at 4 °C.

For depletion of lineage-positive cells, the suspension was incubated 5 min on ice on the depletion magnet until the solution cleared. The entire supernatant was then transferred into a new FACS tube and kept on ice. Then, 1 mL of ice-cold PBS was added to the beads and mixed by vortexing. The depletion step was then repeated, and the two supernatants combined. Then, the cell suspension was centrifuged at 400*g* for 5 min at 4 °C, and the supernatant was removed.

### Isolation of Murine Skin Cells (Mast Cells, Macrophages)

Endogenous mast cells were isolated from Cma1-Cre Rosa26^LSL:Cox8a-Dendra2^ mice, which express a green fluorescent label in skin-resident mast cells. Endogenous macrophages were isolated from Lyz2-Cre Rosa26^LSL:TdTomato^ mice, which express a red fluorescent label in tissue-resident macrophages. The individual mouse strains that were crossed to generate these transgenic mouse strains have been described previously.^34−37^ Cma1-Cre mice were kindly provided by Prof. Axel Roers (Universitätsklinikum Heidelberg, Germany), and other mouse strains were obtained from Jackson Laboratories (Cat# 018385, 007914, 004781). Mice were handled and euthanized according to guidelines and animal protocols approved by the German authorities. Afterward, the back fur was removed by depilation cream. The entire dorsal skin of each mouse was cleaned, washed, and carefully detached from the underlying tissue to avoid contamination with adipose tissue. Mouse ears were separated into dorsal and ventral parts and were, together with the back skin, finely minced in 1.5 mL of Opti-MEM (Gibco, Cat# 51985-026) on ice using sharp scissors. Afterward, the tissue suspension was equally separated into three 2 mL reaction tubes, and 500 μL of Opti-MEM containing 0.5 mg/mL Liberase TM (Roche, Cat# 0541119001), 2.4 mg/mL collagenase 4 (Abnova, Cat# P5275), and 2 mg/mL DNAse1 (Roche, Cat# 10104159001) was added per sample. Samples were then incubated at 37 °C on a bench-top tube shaker at 1500 rpm for 80 min. Samples were resuspended every 20 min. Afterward, the samples were collected and pooled per mouse on ice in a 50 mL reaction tube containing 20 mL of Opti-MEM. The suspension was filtered through a 70 μm cell strainer (Miltenyi, Cat# 130-098-462) into a fresh 50 mL reaction tube, centrifuged for 5 min at 400*g* at 20 °C, and the resulting cell pellet was washed once using 10 mL of Opti-MEM before the samples were centrifuged once more, and the supernatant was discarded. Erythrocyte lysis was performed for 15 min on ice (buffer contained: 155 mM NH_4_Cl, 10 mM KHCO_3_, 0.1 mM EDTA; in deionized H_2_O), and cells were washed once more with 10 mL of Opti-MEM.

### Isolation of Murine Spleen Cells (B Cells and T Cells)

C57BL6/J mice were handled and euthanized according to guidelines and animal protocols approved by the German authorities. Spleens were removed and kept on ice for the whole procedure. A single-cell suspension of splenocytes was obtained by passing the spleens with PBS through a 70 μm strainer using the thumb rest of a syringe plunger. The cell suspension was centrifuged at 300*g* for 5 min at 4 °C, and the cell pellet was resuspended in 1 mL of ACK lysis buffer (Gibco, Cat# 1049201). Lysis was stopped after 2 min with PBS, and the suspension was centrifuged again at 300*g* for 5 min at 4 °C before discarding the supernatant.

### Cultivation of Jurkat Cells

Jurkat cells were cultivated in RPMI 1640 with 10% FCS, 100 U/mL penicillin, and 0.1 mg/mL streptomycin at 37 °C and 5% CO_2_. Cells were transferred to a fresh medium every 2 to 3 days. Twenty-four hours prior to harvest, cells were transferred to a fresh medium (5 × 10^5^ cells in 2 mL) containing 20 μM etomoxir and 0.4 μL of DMSO. Control cultivations without etomoxir were performed accordingly. Immediately before harvest, the concentration of cells in the suspension was determined by counting with a Neubauer-improved counting chamber. For FACS-based sample preparation, an aliquot of the cell suspension was subjected to FACS sorting.

For centrifugation-based sample preparations, aliquots containing 5000 cells were transferred to 1.5 mL tubes and centrifuged at 250*g* for 5 min at 4 °C. Cells were briefly washed in 9 g/L NaCl_aq_ and centrifuged at 250*g* for 5 min at 4 °C. Subsequently, the supernatant was removed by pipetting, and cell pellets were resuspended in 5 μL of 5 g/L NaCl_aq_ before adding 25 μL of acetonitrile with 0.1% ^13^C internal standard solution. Samples were vortexed and incubated at 20 °C for 20 minutes. Subsequently, samples were centrifuged at 20,000*g* for 3 min at 4 °C to obtain a clear supernatant, which was then transferred to a 96-well PCR plate and sealed with EZPearce heat seal foil. To obtain blank samples, an equal volume of sterile culture medium was processed in the same way.

### FACS Staining

Surface staining of either erylysed blood or splenocyte suspension (for sorting for T cells and B cells) or lineage-depleted cells (for sorting stem/progenitor cells) was performed by resuspending the cell pellet in ice-cold PBS containing the surface antibody cocktail A (CD4-PeCy5 (Cat# 100410, 1:1000), CD8a-PeCy5 (Cat# 100710, 1:2000), B220-AF700 (Cat# 103231, 1:300)) for sorting B and T cells from blood, antibody cocktail B (CD4 (Cat# 100422, 1:1000)/CD8a (Cat# 100722, 1:1000)/B220 (Cat# 103222, 1:1000)/Ter119 (Cat# 116221, 1:500)/Gr-1 (Cat# 108416, 1:500)/CD11b (Cat# 101216, 1:1000)-all PeCy7, c-Kit/CD117-PE (Cat# 105808, 1:1000), Sca-1-APC-Cy7 (Cat# 108126, 1:500), CD150-BV605 (Cat# 115927, 1:200), CD48-BV421 (Cat# 103428, 1:500)) for sorting stem/progenitor cells, or antibody cocktail C (CD3-PE (Invitrogen Cat# 12-0031-83, 1:200), B220-A647 (Cat# 103226, 1:200)) for sorting B and T cells from spleen, followed by incubation for 30 min at 4 °C in the dark. All antibodies were purchased from BioLegend unless noted otherwise. Mast cells and macrophages were stained with the Zombie NIRTM fixable viability dye (BioLegend, Cat# 423105; 1:200) in 500 μL of Opti-MEM per sample for 10 min at 20 °C in the dark.

Cells were washed with 1 mL of ice-cold PBS and centrifuged at 400*g* for 5 min at 4 °C, and the supernatant was then discarded. Depending on the size of the pellet, the cells were resuspended in an appropriate volume of ice-cold physiological water (9 g/L NaCl, usually between 500 μL and 2 mL) containing a 1:200 ratio internal standard stock. The internal standard stock was 6 mg/mL chloro-phenylalanine and 6 mg/mL aminoterephthalic acid in 30% v/v 2-propanol in water. Finally, cells were filtered through a 30 μm cell strainer (mast cells and macrophages) or filter-cap FACS tube (all other cell types) to immediately proceed with flow cytometry-based sorting.

### Preparation of the Sorting Plate

For the collection of 5000 cells, 25 μL of extraction solution was added in each well of an Eppendorf twin.tec LoBind clear skirted PCR 96-well plate and immediately covered with a PCR lid (stripes cut into singles) to avoid evaporation. Extraction solution contained 0.1% 13C internal standard stock (1 aliquot of U-^13^C yeast extract (Isotopic solutions, Cat# ISO-1) in 10 mL of 25% v/v methanol in water) in acetonitrile (LC-MS grade). For lower cell numbers, a corresponding amount of sheath fluid was added to ensure constant ratios of organic solvents and water in all samples (Table 1).

**Table 1:** Pipetting Scheme for Sorting of Different Number of Cells^a^

cell number	extraction solution (μL)	5 g/L NaCl in water (μL)	total volume (μL)
5000	25	0	25
4000	25	1	26
3000	25	2	27
2000	25	3	28
1000	25	4	29
500	25	4.5	29.5
100	25	4.9	29.9

a Volumes are given per well.

### Setup of FACS Aria III for Cell Sorting

Cell sorting was carried out on a BD FACS Aria III. The analysis of data was performed using BD FACSDiva software. The sorter was configured with a 70 μm nozzle tip and lasers at 405, 488, 561, and 633 nm. Sheath pressure was set to 70 psi, and sheath fluid was composed of 5 g/L NaCl in deionized water. Sort Mode was set to 4-way purity (Yield Mask 0, Purity Mask 32, and Phase Mask 0). The drop frequency was set to 90400 Hz (amplitude of 17.9 and drop delay at 46.76). The plate voltage was set to 5000 Volts. The markers used to sort cell populations are listed in Table 2, the detection filter sets are listed in Supporting Table S1, and the applied sorting gates can be seen in Supporting Figures S1–S4. Jurkat cells were sorted on forward-scatter and sideward-scatter signals to select single cells.

**Table 2:** Markers Defining the Different Cell Populations

cell type	markers
B cells	B220+
T cells from blood	CD4+CD8a+
T cells from spleen	CD3+
mast cells	Cma1-Cre (MitoDendra)+Zombie NIR-
macrophages	Lyz2-Cre (TdTomato)+Zombie NIR-
LSK (lineage-c-kit+Sca-1+) cells	CD4-CD8a-CD11b-B220-GR-1-Ter119-c-Kit+Sca-1+
HSCs (hematopoietic stem cells)	CD4-CD8a-CD11b-B220-GR-1-Ter119-c-Kit+Sca-1+CD150+CD48-
MPPs (multipotent progenitors)	CD4-CD8a-CD11b-B220-GR-1-Ter119-c-Kit+Sca-1+CD150-CD48+

Cells were sorted at a flow rate of less than 15,000 events per second to obtain high efficiency of sorting. In this context, events are droplets generated in the cell sorter that contain a detectable light-scattering signal. An equal number of droplets containing cells of interest or droplets containing a very low light-scattering signal (likely cell debris) were collected. To minimize evaporation of extraction solution, the automated cell deposition unit (ACDU) holding the 96-well plate was cooled to 4 °C and the lids on the 96-well plate were only removed while cells or debris was deposited into the respective wells.

PCR plates with cells in extraction solution were centrifuged for 10 min at 1600*g*. Subsequently, 25 μL of clear supernatant was transferred to a fresh PCR plate that was then sealed with EZPearce heat seal foil.

### Timing

The time required to perform sample preparation from the intact organ or cell culture all the way to the cell extract depends on the number of replicates that are prepared in parallel, the experience of the person performing the experiment, and the sample preparation protocol at hand. We estimate the times that were required for preparing the samples for this work as follows: peripheral blood cells: 60 min, stem cells and progenitor cells from bone marrow: 5 hours, tissue-resident immune cells from skin: 4 hours, and splenocyte and cultured Jurkat cells: 30 min.

### LC-QQQ-MS Measurement and Data Preprocessing

Whenever samples could not be measured within a few hours, they were stored at −80 °C. Prior to LC-MS analysis, stored samples were briefly thawed and then sonicated for 5 min to ensure complete resuspension of metabolites that might have precipitated during storage.

Targeted metabolite quantification was modified from a published method.^38^ An Agilent 1290 Infinity II UHPLC was coupled to an Agilent 6495 QQQ-MS operating in MRM mode. MRM settings were optimized separately for all compounds using pure standards. For every target metabolite, one quantifier and 1 to 2 qualifiers were recorded. Chromatographic separation was performed using a HILICON iHILIC(P) classic column (100 mm × 2 mm, 5 μm particles) or a Waters Atlantis Premier BEH ZHILIC column (100 mm × 2.1 mm, 1.7 μm particles). Buffer A was 20 mM ammonium carbonate and 5 μM medronic acid in Milli-Q H_2_O and Buffer B was 90:10 acetonitrile/buffer A. The gradient profile was: 0 min, 95% B, 120 μL/min; 18 min, 55% B, 120 μL/min; 19 min, 20% B, 120 μL/min; 21.5 min, 20% B, 120 μL/min; 22 min, 95% B, 120 μL/min; 23.5 min, 95% B, 120 μL/min, 25.5 min, 95% B, 300 μL/min; stop time 30 min. Irrespective of the BEH ZHILIC column used, the flow rate was increased to 150 μL/min from 0 min to 23.5 min. The injection volume was 20 μL, the column temperature was 40 °C, and the autosampler temperature was 5 °C. MS source parameters were as follows: gas temp: 240 °C, gas flow: 15 L/min, nebulizer: 50 psi, sheath gas temp: 400 °C, sheath gas flow: 11 L/min, capillary voltage: 2000 V, and nozzle voltage: 300 V. iFunnel parameters were as follows: high-pressure RF positive: 110 V, high-pressure RF negative: 90 V, low-pressure RF positive: 80 V, and low-pressure RF negative: 60 V.

Raw data in .d format was converted to .mzML format using ProteoWizard^39^ and subsequently preprocessed using the R package automRm.^38^ Metabolites that were detected in less than 10 samples were removed from the data set. Signal intensities were normalized to ^13^C-labeled internal standards using the best-matched internal standard approach,^40^ followed by missing number imputation (half-minimum value of the respective metabolite) and the removal of outliers by *z*-score filtering.

## Results and Discussion

### Impact of Sheath Fluid

HILIC chromatography is sensitive to salts contained in samples, particularly to pH buffers. However, the sheath fluid used in cell sorters must contain ions to enable deflection of droplets to the desired position in the sample collection device. Solutions of NaCl in water have a lower pH buffer capacity compared to PBS and therefore hold the potential to cause less problems in HILIC chromatography.

We have observed the stability of the deflection of droplets into side streams using 0, 1, 2, and 5 g/L NaCl in deionized water by visual inspection of the streams in the side stream camera view. At 0 g/L, no deflection of droplets was possible. At 1 g/L, the side streams were highly unstable; at 2 g/L, the side streams were mostly stable; and at 5 g/L, the side streams were as stable as with PBS as the sheath fluid.

**Figure 2:**
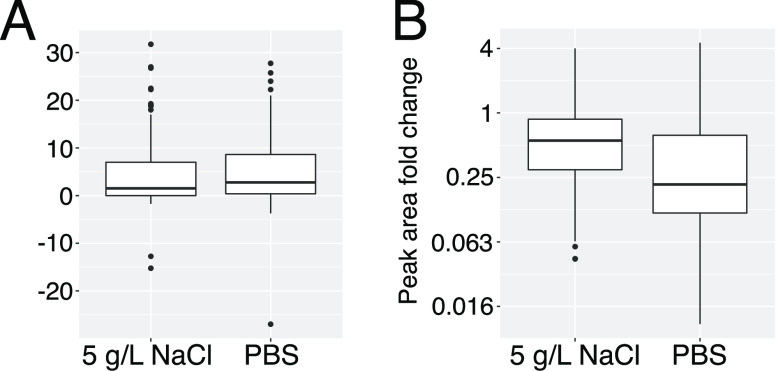
(A) Shift in the retention time and (B) difference in signal intensity caused by the addition of 5 g/L NaCl or PBS compared to a water reference for 111 metabolites analyzed by HILIC chromatography.

We have compared the impact of the commonly used sheath fluid phosphate-buffered saline (PBS) to that of sodium chloride solution (5 g/L NaCl in deionized water) on shifts in retention time (RT) and changes in the peak area (Figure 2). To this end, we have prepared a mix of 111 metabolites (including amino acids, organic acids, nucleotides, cofactors, and acylcarnitines). To simulate the use of different sheath fluids, we have added 4 μL of buffer and 25 μL of acetonitrile to 1 μL of metabolite mix, where the buffer was either 5 g/L NaCl in deionized water, PBS, or Milli-Q H_2_O (as a reference).

PBS caused slightly more pronounced shifts in RT compared to 5 g/L NaCl (Figure 2A). Most peaks shifted toward increased RT. Shifts in RT can make the correct integration of chromatographic peaks difficult, in particular for automatic peak detection algorithms. Larger shifts in RT might even cause metabolites to elute outside the predefined detection window of targeted LC-MS methods and thus not be detected.

PBS caused more pronounced differences in signal intensity compared to 5 g/L NaCl (Figure 2B). Most peak intensities were reduced in the presence of the buffers, pointing toward ion suppression and formation of adducts as possible causes for this reduction. Differences in signal intensity can sometimes be tolerated if all samples of a batch are affected in the same way or if the differences can be compensated using internal standards. However, strongly reduced signals might fall below the limit of detection of the applied LC-MS method and thus cause metabolites to be lost from a data set.

PBS had a stronger potentially negative impact on the detection of our diverse panel of metabolites. Therefore, we have opted to use 5 g/L NaCl as sheath fluid for subsequent experiments.

### Background Control Samples

For the quantification of intracellular metabolites in very small amounts of sample material, it is particularly important to ensure that the measured signal originates from the cells in the sample. Other sources of signals include the buffer in which the cells are suspended when entering the sorter, contamination from the sheath fluid, the extraction buffer, and from the containers and pipette tips that were used to handle the samples.

Contaminations from the extraction buffer and the sheath fluid can be highly relevant because they contribute to the bulk of the sample that is loaded onto the LC-MS. For the sort conditions used in this study, we have measured the average droplet volume to be 1 nL. Based on typical consumption rates of cell suspension and sheath fluid, we have estimated that droplets contain a 1000:1 ratio of sheath to sample. For a prototypic cell, we assume a diameter of 10 μm and a spherical shape. Based on these numbers, we have estimated cells to contribute less than 0.01% of the volume of the sample (Table 3). To evaluate the reproducibility of the number of sorted cells, we have sorted 10 times 100 events containing beads onto glass slides and then counted the number of beads on the slide. On average, 100 events correspond to 95 beads with a standard deviation of 1.94.

**Table 3:** Estimated Sizes of Sample Components

	diameter of sphere (μm)	volume per droplet (nL)	volume in sample of 5000 droplets (nL)
cells	10	0.0005	2.5
cell suspension buffer	12	0.0005	2.5
sheath fluid	124	0.999	4995
total droplet	124	1	5000
extraction solution			25,000
total sample			30,000

To obtain blank samples that contain all the same contaminations as the cell extracts, we have sorted the same number of events with very low forward- and sideward-scatter signals, assuming that these droplets do not contain intact cells (Supporting Figures S1–S4). However, these samples can still contain cell debris. For technical reasons, it was impossible to sort droplet void of any detectable signal.

**Figure 3:**
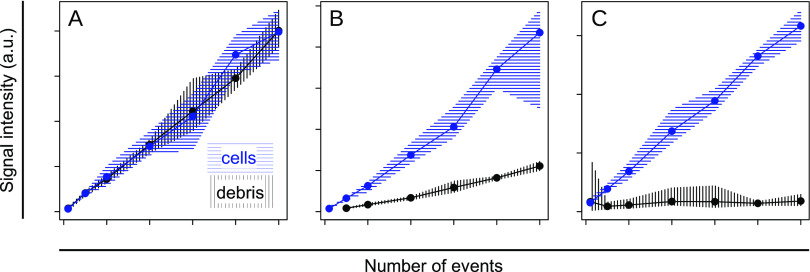
Signal intensity over a number of sorted events for debris (black) and B cells from spleen (blue) for (A) aminoterephthalic acid (ATA, left panel), (B) S-adenosyl-methionine (SAM), and (C) 4-aminobutyric acid. Solid lines and dots indicate the mean, and the hatched area indicates the min-to-max range (*n* = 8). ATA had been spiked into the cell suspension before cell sorting and thus represented an example of an extracellular compound. A sizable fraction of SAM occurs outside the cells, but the amount detected in intact cells is still larger. 4-Aminoburyric acid occurs almost exclusively inside the cells.

To test the contribution of the cell suspension buffer to the final sample, we monitor the signal intensity of aminoterephthalic acid (ATA), which has been added to the cell suspension prior to sorting, across samples with a different number of events (Figure 3). We have selected ATA because this compound is highly polar, and, to the best of our knowledge, there are no known biochemical degradation reactions. Moreover, ATA exhibited good chromatographic retention and a solid MS signal in the employed LC-MS method. Indeed, the signal intensity of ATA is virtually identical between debris events and events containing cells across all tested number of events. In contrast, 4-aminobutyric acid, a representative example of intracellular metabolites, has close to zero signal intensity in all debris samples and correlates well with the number of events for cell samples. S-adenosyl-methionine (SAM) is an example of a metabolite that occurs in debris samples but nevertheless is detected in cells at higher levels. In summary, we propose that samples containing debris events are more realistic “background” control samples than water or sheath fluid.

### Effects of FACS-Based Sample Preparation

**Figure 4:**
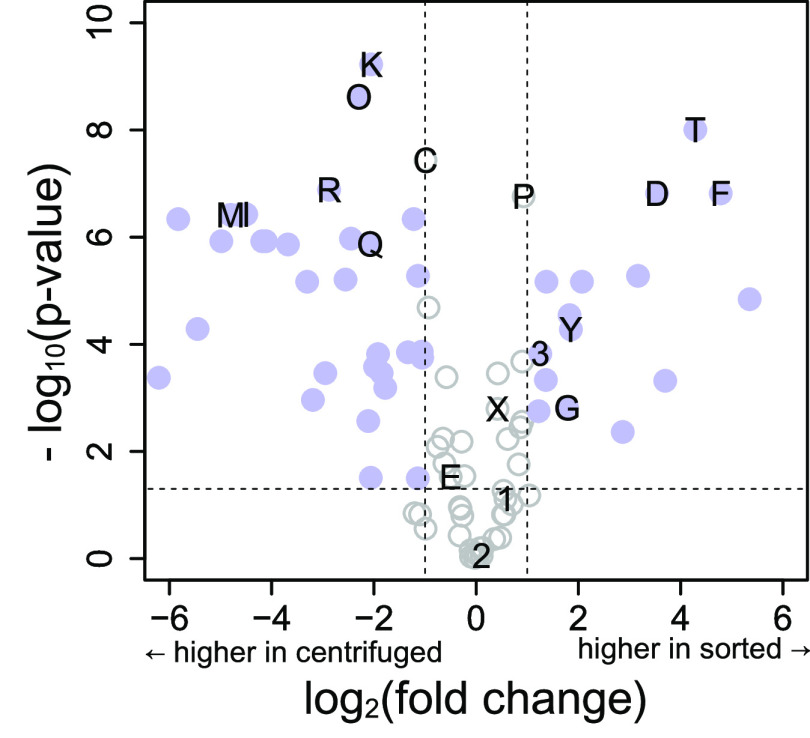
Volcano Plot of differences between Jurkat cells harvested by centrifugation (5000 cells, *n* = 8) or by our FACS-based protocol (5000 events, *n* = 8). Vertical dashed lines indicate a fold change of ±2, and the horizontal dashed line indicates a *p*-value of 0.05 (two-sided t-test, unequal variance). The 46 metabolites with a fold change greater than ±1 and a *p*-value of less than 0.05 are indicated by blue solid circles, and the other 40 metabolites are indicated with gray open circles. Selected metabolites are labeled: 3: adenosine triphosphate (ATP), 2: adenosine diphosphate (ADP), 1: adenosine monophosphate (AMP), D: dihydroorotic acid, E: glutamic acid, F: flavin-adenosine-dinucleotide (FAD), G: glutathione (GSH), I: isoleucine, K: lysine, M: methionine, O: ornithine, P: proline, Q: glutamine, R: arginine, T: thiamine, X: oxidized nicotinamide-adenine-dinucleotide (NAD), and Y: reduced nicotinamide-adenine-dinucleotide (NADH).

As the metabolome of cells in tissue prior to sample preparation is unknown, the effect of FACS-based sample preparation cannot be directly determined. Therefore, we used Jurkat cells (a cell line that grows in suspension culture) to compare the FACS-based sample preparation to a more established centrifugation-based sample preparation method (Figure 4). Aliquots of 5000 cells were generated from the same culture using both protocols. Only metabolites were included that were significantly above blank in data from both protocols (FDR < 0.05, calculated by the Mann–Whitney signed rank test, corrected for multiple testing by the Benjamini & Hochberg method).

Many differences can be observed in the metabolomes generated with FACS-based sample preparation and centrifugation-based sample preparation. Metabolites that are significantly higher in centrifuged samples include several amino acids, which are present at high concentrations in the cultivation medium. We have estimated that a FACS-sorted debris sample contains 5 nL of cultivation medium. Using phenol red (a pH indicator that is commonly contained in cultivation medium) as a proxy for the medium content, we have calculated that samples generated by the centrifugation-based protocol contain, on average, 270 nL of the carried-over medium. This indicates that for samples with low cell numbers, the separation of cultivation medium from cells is more stringent with FACS-based sample preparation.

The ratio of the “energy currency” metabolites ATP and ADP has previously been used as an indicator for the quality of sample preparation.^26^ Contrary to previous work, we find this ratio to be higher in samples prepared by the FACS-based protocol than in samples prepared by a centrifugation-based protocol, indicating more rapid quenching of metabolism in our FACS-based samples. A major difference between this work and previous work is the number of cells per sample. Sorting of 5000 events takes less than 1% of the time required for sorting 6 × 10^5^ events from the same cell suspension. By comparison, centrifugation-based sample preparation takes almost the same time for both cell numbers. This indicates that FACS-based sample preparation can be advantageous for samples with low cell numbers.

**Figure 5:**
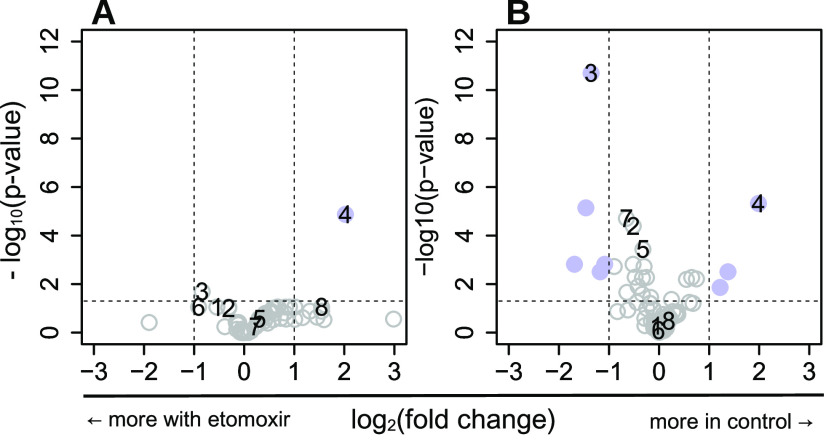
Volcano plots of differences between etomoxir-treated (*n* = 8) and control (*n* = 8) Jurkat cells. (A) 5000 cells were harvested by centrifugation. (B) 5000 events were collected with our FACS-based protocol. Vertical dashed lines indicate a fold change of ±2, and the horizontal dashed line indicates a *p*-value of 0.05 (two-sided t-test, unequal variance). Metabolites with a fold change greater than ±1 and a *p*-value of less than 0.05 are indicated by blue solid circles, and all other metabolites are indicated with gray open circles. Selected metabolites are labeled: 1: proline, 2: carnitine, 3: propionyl-carnitine, 4: butyryl-carnitine, 5: S-adenosyl-homocysteine, 6: S-adenosyl-methionine, 7: guanosine-diphosphate, and 8: adenosine triphosphate.

To test if the same biological conclusions can be derived from samples prepared by our FACS-based sample protocol and a centrifugation-based protocol, we have performed parallel experiments in which Jurkat cells were exposed to 20 μM etomoxir (a carnitine palmitoyltransferase I inhibitor) for 24 h prior to sampling (Figure 5). Only metabolites were included that were significantly above blank in both etomoxir-treated and control samples (FDR < 0.05, calculated by the Mann–Whitney signed rank test, corrected for multiple testing by the Benjamini & Hochberg method). In both data sets, the most significantly increased metabolite in the presence of etomoxir is propionyl-carnitine and the most significantly decreased metabolite is butyryl-carnitine. This indicates that the major effects of etomoxir treatment are conserved in both sample preparation protocols.

### Number of Detected Metabolites Depends on the Cell Number

**Figure 6:**
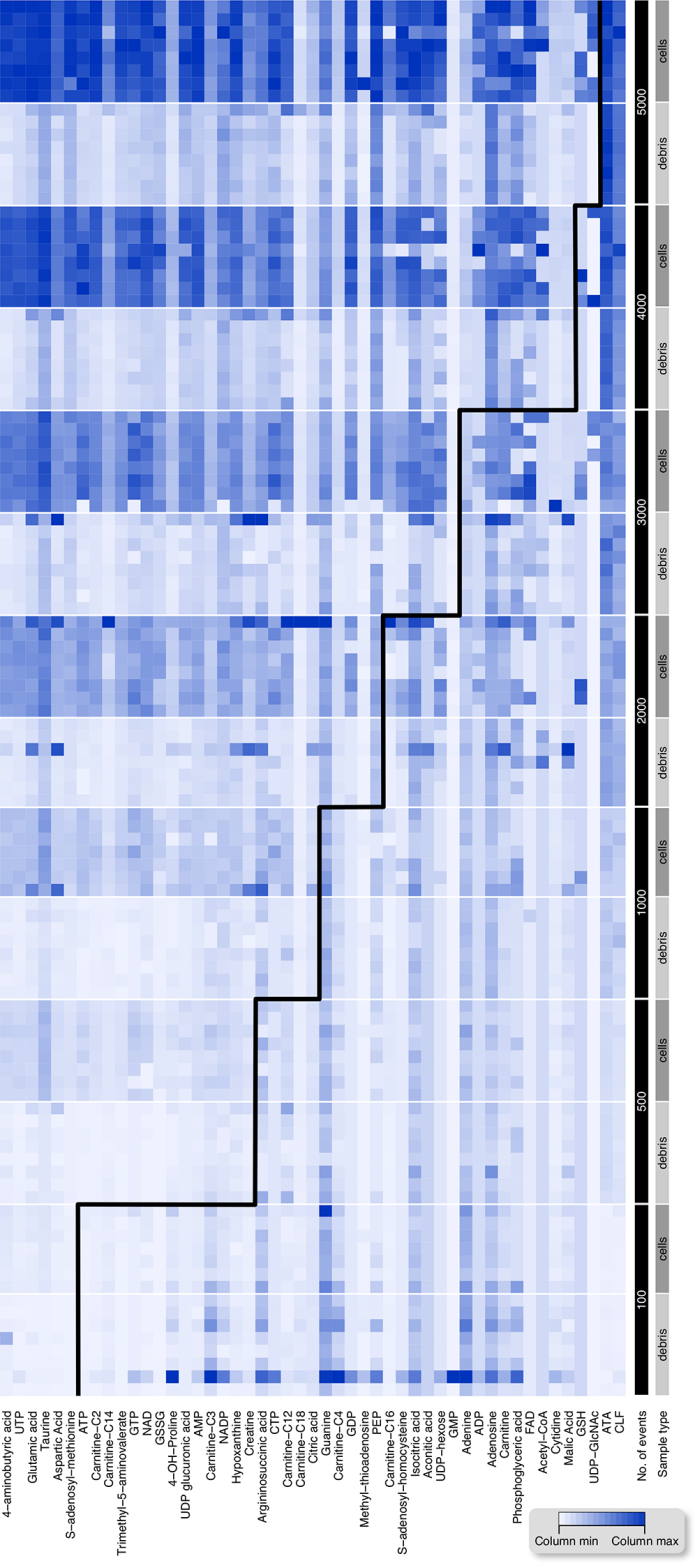
Heatmap of series of extracts of B cells from spleen and matching debris samples with a varying number of sorted events. A total of 8 replicates per group were generated, but 3 outlier samples were detected by *z*-score filtering and removed. Above the black line are samples in which the signal intensity in cell extracts is significantly larger than in debris (FDR < 0.05 determined by a one-sided Wilcoxon rank sum test corrected for multiple testing with the Benjamini & Hochberg method). The internal standards ATA and CLF were included, but 28 metabolites that were detected but not above the blank level were omitted from the plot.

To characterize the relationship between the number of extracted cells and the number of metabolites that could be detected above the background level, we have sorted 8 aliquots, each containing 100 to 5000 B cells or debris events from a suspension of murine spleen cells (Figure 6). Three outlier samples were detected by *z*-score filtering (abs(*z*-score) > 3) on the event number-normalized data. Subsequently, we have defined a given metabolite in a given number of events to be detected above background if the signal intensity in cell extracts was significantly larger in cell extracts than in debris samples (FDR < 0.05 determined by a one-sided Wilcoxon rank sum test corrected for multiple testing with the Benjamini and Hochberg), and this metabolite has also been detected above background in the next higher number of events. With this definition, we observed a monotonously increasing number of detected metabolites with an increasing number of events in a range from 6 detected metabolites in 100 events up to 47 detected metabolites in 5000 events. The same trend could also be observed for T cells from the spleen and LSK cells (Supporting Figures S6 and S7).

### Cell-type-Specific Differences

To test if the workflow is applicable to cell types other than splenic B cells and T cells, we have subjected 6 very different cell types sorted from 3 different tissues to the same workflow. Only the generation of cell suspensions, fluorescent staining, and FACS gating were adapted to each cell type. The cell types include stem/progenitor cells isolated from bone marrow (HSCs, MPPs), cells isolated from peripherical blood (B cells, T cells), and cells isolated from the skin (macrophages, mast cells).

**Figure 7:**
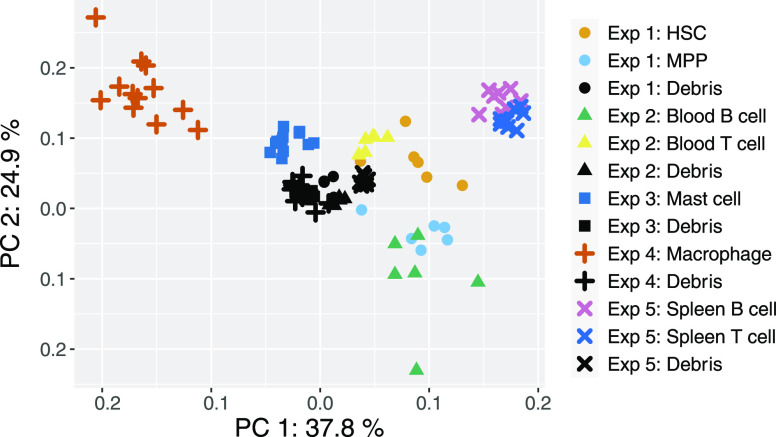
Principal component analysis of metabolic profiles obtained for 8 different cell types in 5 different experiments using an input of 5000 events per replicate. Only 10 metabolites that were detected above background in all cell types were included in this analysis. Colors indicate the cell type, and symbols indicate the experiment. Debris (background) samples from all experiments lie close together in the center of the plot, while all cell types are clearly separated. The number of replicates is 6 in experiments 1 and 2, 12 in experiments 3 and 4, and 8 in experiment 5.

Cell-type-specific differences were preserved across a broad range of diverse metabolites (Figure 7, Supporting Figure S8). A total of 80 metabolites were detected above background in at least one cell type, while 10 metabolites were detected above background in all 8 cell types. Moreover, the number of metabolites that were detected above background differed between cell types from 18 (T cells from the blood) to 57 (macrophages). Possible reasons for the observed difference in the number of detected metabolites include differences in cell size, in the environmental stress experienced during the different sample preparation protocols, in susceptibility to sorting-induced cellular stress, or in the metabolic state of the cells. Of note, the distance of cell types to debris (background) samples in the PCA plot does not correlate with the number of metabolites that were detected above the background. For example, HSCs and MPPs (28 detected metabolites each) are located further away from the background samples in the PCA plot than blood T cells (18 metabolites) and mast cells (51 metabolites). The differences between B and T cells from the spleen and the same cell types from the blood might reflect the effect of the different sample preparation strategies that were required for these two tissues.

**Figure 8:**
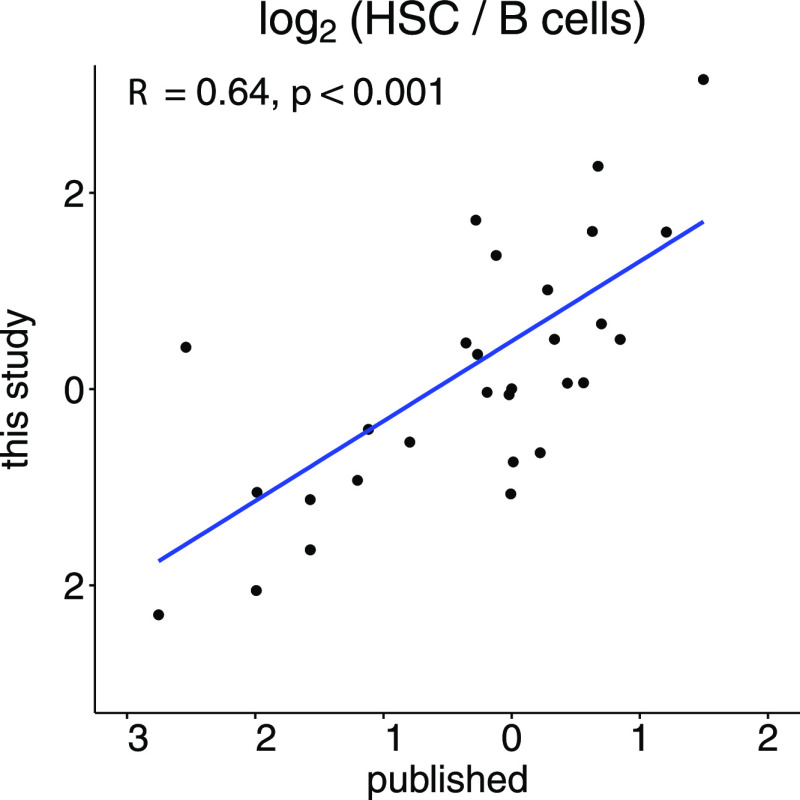
Correlation of ratio of the median signal intensity between HSCs and B cells comparing the published data and this study. A total of 30 metabolites were measured in both studies.

Each cell type exhibits a characteristic metabolic profile, indicating that cell-type-specific differences were conserved during sample preparation. Moreover, the metabolic profiles of the debris samples also differ among the different cell sorts, indicating that sample-type-specific differences are reflected in these samples and underlining the importance of using these relevant blank reference samples. To compare our data to published data,^41^ we correlated the logarithm of the ratio of the median signal intensity in HSCs and B cells, the two cell types that were used in both studies (Figure 8). Note that the published data was only available in the auto-scaled form, which can impact the magnitude of the derived ratios. A total of 30 metabolites were measured in both studies, and we found a good agreement (*R* = 0.64, *p* < 0.001) between both data sets.

## Conclusions

In this work, we present a comprehensively optimized workflow that is optimized for samples of 5000 cells but enables the targeted detection of intracellular metabolites in as little as 100 FACS-sorted cells. The coordinated optimization of the workflow allows the highly sensitive detection of metabolites using standard hardware that is robust enough for routine use. For example, the addition of internal standards to the cell suspension and to the extraction solution facilitates troubleshooting and allows normalization of technical variations. Moreover, changing the sheath fluid for the sorting process from the widely used PBS to 5 g/L NaCl reduces the load of inorganic ions and the pH buffer capacity of the samples, both of which have previously been described to negatively impact HILIC chromatography. A final concentration of 80% v/v acetonitrile has successfully been used for metabolite extraction in the past,^31,42,43^ and at the same time allows a large injection volume on the LC-MS.

A small amount of the buffer in which cells were suspended for FACS sorting will be carried over to the cell extract. We estimate this volume to be about 2.5 nL for 5000 events. In the alternative sample preparation tested for Jurkat cells that isolates cells by centrifugation and manual pipetting of supernatant, this carry-over volume was found to be more than 100-fold larger. Nevertheless, even a small amount of carry-over can be a source of the background signal, in particular for metabolites that are present in high extracellular concentrations in the cell suspension. Additional sources of the background signal can be carried over in the LC autosampler or column memory effects. In our hands, the latter can be observed for late-eluting compounds such as basic amino acids. In common, all sources of carry-over can restrict the number of metabolites that can be meaningfully interpreted.

The turnover of some metabolites can change within seconds in response to environmental perturbation.^44^ Consequently, any sample preparation protocol in which quenching of metabolism takes longer than these few seconds will lead to some sort of sampling artifacts. Therefore, data obtained with our FACS-based protocol can differ from data obtained with other protocols (and from the unknown “true” metabolic state prior to sampling). The advantages of our FACS-based protocol compared to a classical centrifugation-based protocol become more pronounced when the number of cells per sample is lower. The reason is that in the FACS-based protocol, but not in the centrifugation-based one, medium carry-over and time required for sorting scale linearly with the number of sorted events. Sorting cells directly into extraction buffer avoids common sources of cell number variations, such as cells sticking to collection tubes. This enables a good reproducibility of the number of cells per sample.

Any type of experimental artifact must be taken into account when interpreting the obtained data. Biologically relevant phenotypes can be observed despite the possible presence of artifacts and thus render these experiments useful.

We have focused on polar metabolites because they are of particular importance to many research projects in our laboratories. However, the number of detected compounds can be further increased, for example, by the addition of polar lipids to the LC-MS method. Previously, the separation of phospholipids on zwitterionic HILIC columns has been demonstrated.^45^ Moreover, in initial tests with the same chromatography for discovery metabolomics on an LC-QTOF setup, we have found phospholipids, including phosphocholines, phosphoinositols, and phosphoethanolamines, to elute in the first 2 min and lysophospholipids to elute in between 2 and 3 min. Additional polar metabolites can also be added to the method; however, since we require pure standards for the optimization of compound-specific LC-QQQ-MS settings, this is a costly endeavor.

Broad coverage of diverse metabolites in a single analysis by zwitterionic HILIC renders the described method useful for many different scientific questions. Targeted metabolomics using internal standards and multiple MRM transitions for every target molecule ensure high data quality. The use of normal flow LC provides the robustness that allows the implementation of this protocol on LC-MS machines that are used for diverse chromatographic methods. The omission of drying or derivatization circumvents possible introduction of experimental errors. Reducing the number of cells required for generating a meaningful metabolomic readout has the potential to reduce the number of animals required for projects involving rare cell populations and to maximize the insight that can be gained from cells isolated from patient samples.

## Supporting Information Available

Filter sets used for the isolation of different cell populations (Table S1); gating strategy for obtaining separated cell populations in FACS sorting (Supporting Figures S1–S5); heatmap of series of extracts of T cells from spleen (Supporting Figure S6); heatmap of series of extracts of LSK cells (Supporting Figure S7); and heatmap of metabolite profiles of 8 different cell types (Supporting Figure S8) (PDF).

## Notes

Ethical review and approval were not required for this study in accordance with §4 (3) of the German Animal Protection Act.
