# Supplementary material for: LC-MS-Based Targeted Metabolomics for FACS-Purified Rare Cells
Source: Anal Chem. 2023 Feb 22;95(9):4325–34. doi: 10.1021/acs.analchem.2c04396 (PMC9996616; doi:10.1021/acs.analchem.2c04396)
Supplement: Supplementary file 1 — ac2c04396_si_001.pdf [file ac2c04396_si_001.pdf]

Supporting Information

LC-MS-based targeted metabolomics for FACS-purified rare cells

Katharina Schönberger[1,2,3], Michael Mitterer[1], Katharina Glaser[1,2,3], Manuel Stecher[1,3,4], Sebastian Hobitz[1], Dominik Schain-Zota[1], Konrad Schuldes[1], Tim Lämmermann[1], Angelika S. Rambold[1], Nina Cabezas-Wallscheid[1], and Joerg M. Buescher[1]*

[1] Max Planck Institute of Immunobiology and Epigenetics, Stübeweg 51, 79108 Freiburg, Germany

[2] International Max Planck Research School for Immunobiology, Epigenetics and Metabolism (IMPRS-IEM), Freiburg, Germany

[3] Faculty of Biology, University of Freiburg, Freiburg, Germany

[4] International Max Planck Research School for Immunobiology, Epigenetics and Metabolism (IMPRS-MCB), Freiburg, Germany

* Corresponding Author: buescher@ie-freiburg.mpg.de

Table of Content

Supplementary Table S 1: Filter sets used for the isolation of different cell populations. S2
Supplementary Figure S 1: Gating strategy to obtain separated populations of LSK, HSC, and MPP. S3
Supplementary Figure S 2: Gating strategy to obtain separated populations of B cells and T cells. S3
Supplementary Figure S 3: Gating strategy to obtain separated populations of macrophages.......................................S4
Supplementary Figure S 4: Gating strategy to obtain separated populations of mast cells...S4
Supplementary Figure S 5: Gating strategy to obtain separated populations of B cells and T cells from spleen. S4
Supplementary Figure S 6: Heatmap of series of extracts of T cells from spleen...S5
Supplementary Figure S 7: Heatmap of series of extracts of LSK cells...S6
Supplementary Figure S 8: Heatmap of metabolite profiles of 8 different cell types. S7

Supplementary Table S 1: Filter sets used for the isolation of different cell populations.

	**B-/T-cells from blood**	**LSK**	**HSC and MPP**	**Mast cells**	**Macro-phages**	**B-/T-cells from spleen**
**BV421**	450/50 BP		450/50 nm BP			
**BV605**	635LP and 660/20 BP		635LP and 660/20 BP			
**FITC**	502LP and 530/30 BP			502LP and 530/30 BP		
**PE**	570 LP and 585/15 BP	570 LP and 585/15 BP	570 LP and 585/15 BP			570 LP and 585/15 BP
**PE-Cy 7**	735 LP and 780/60 BP	735 LP and 780/60 BP	735 LP and 780/60 BP			
**Alexa Fluor 647**						660/20 BP
**Alexa Fluor 700**	690 LP and 730/45 BP					
**APC-Cy 7**	755 LP and 780/60 BP	755 LP and 780/60 BP	755 LP and 780/60 BP	755 LP and 780/60 BP	755 LP and 780/60 BP	
**Tomato**					600 LP and 610/20 BP	

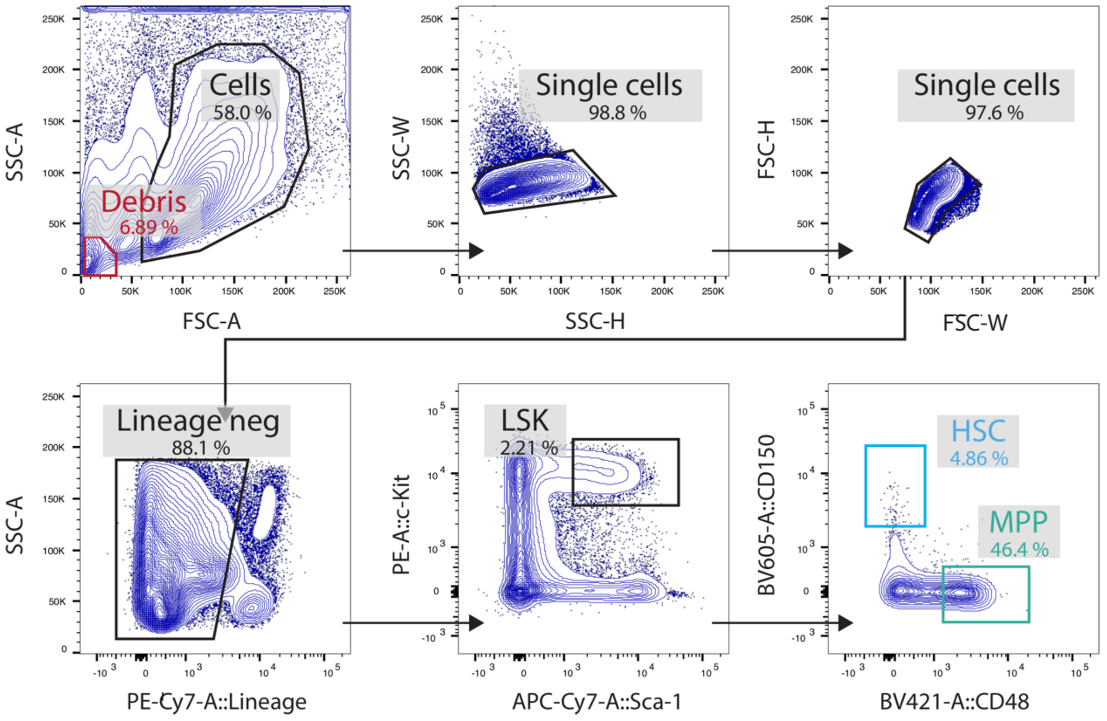

Supplementary Figure S 1: Gating strategy to obtain separated populations of LSK, HSC, and MPP. The sorted populations represent only 1.9% (LSK), 0.87% (MPP) and 0.091% (HSC) of all cells in the input.

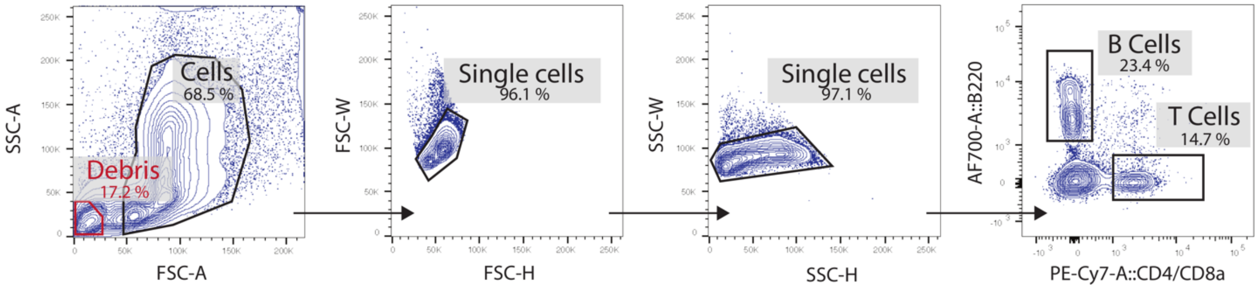

Supplementary Figure S 2: Gating strategy to obtain separated populations of B cells and T cells.

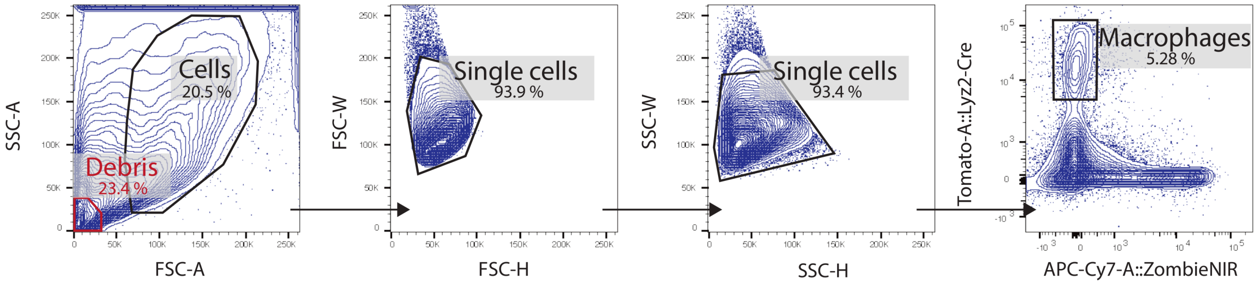

Supplementary Figure S 3: Gating strategy to obtain separated populations of macrophages.

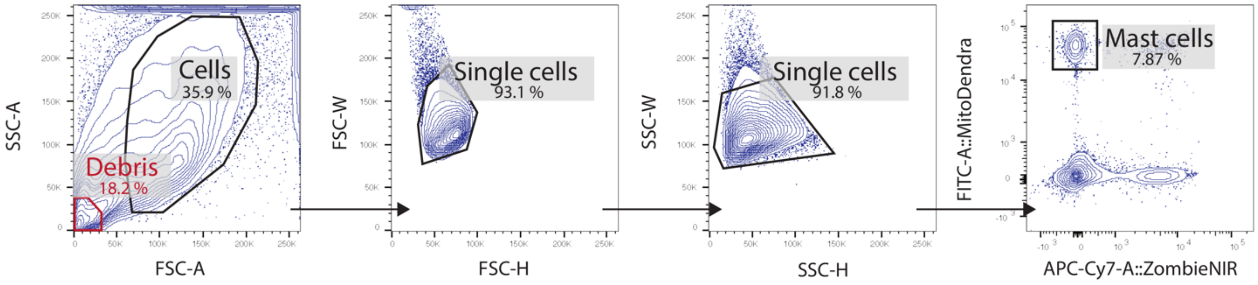

Supplementary Figure S 4: Gating strategy to obtain separated populations of mast cells.

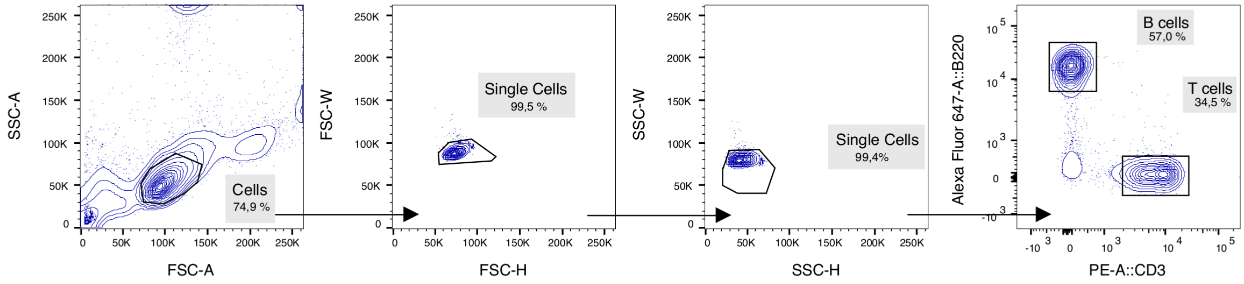

Supplementary Figure S 5: Gating strategy to obtain separated populations of B cells and T cells from spleen.

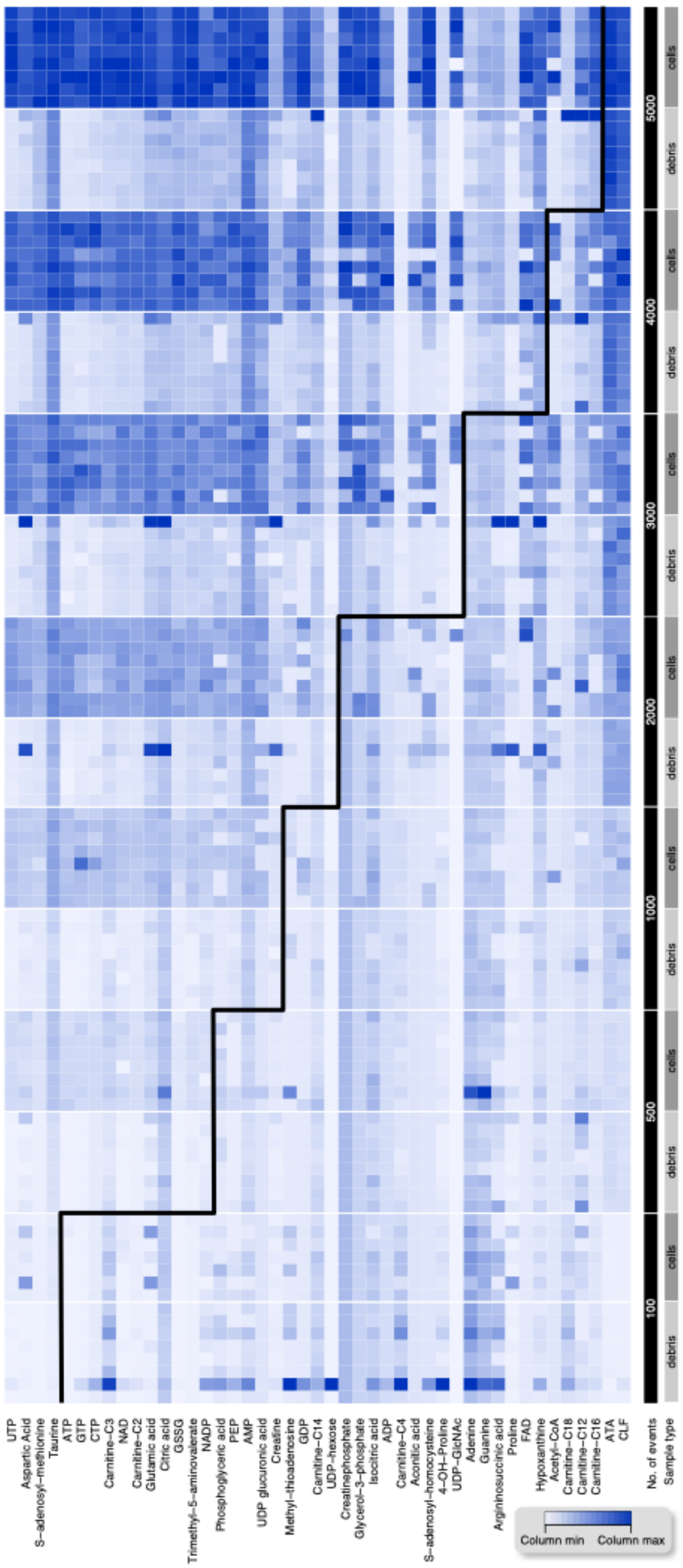

Supplementary Figure S 6: Heatmap of series of extracts of T cells from spleen and matching debris samples with varying number of sorted events. Above the black line are samples in which the signal intensity in cell extracts is significantly larger than in debris (FDR < 0.05 determined by one-sided Wilcoxon rank sum test corrected for multiple testing with the Benjamini & Hochberg method). The internal standards ATA and CLF were included, but 32 metabolites that were detected but not above blank level for any number of events were omitted from the plot.

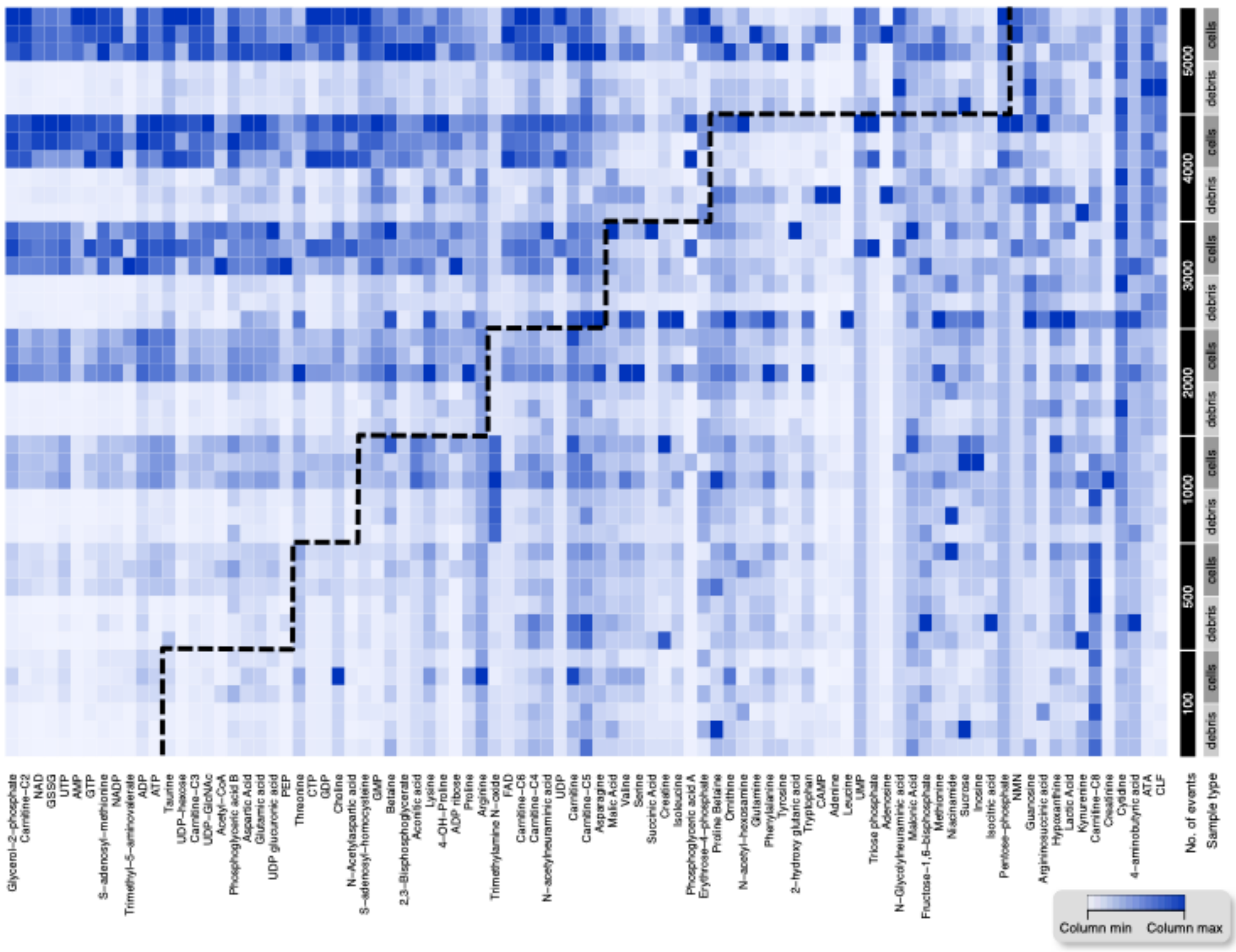

Supplementary Figure S 7: Heatmap of series of extracts of LSK cells and matching debris samples with varying number of sorted events. Above the dashed black line are samples in which the signal intensity in cell extracts is significantly larger than in debris with an FDR < 0.1 determined by one-sided Wilcoxon rank sum test corrected for multiple testing with the Benjamini & Hochberg method. Note that no metabolite at any tested number of events was detected with an FDR < 0.05.

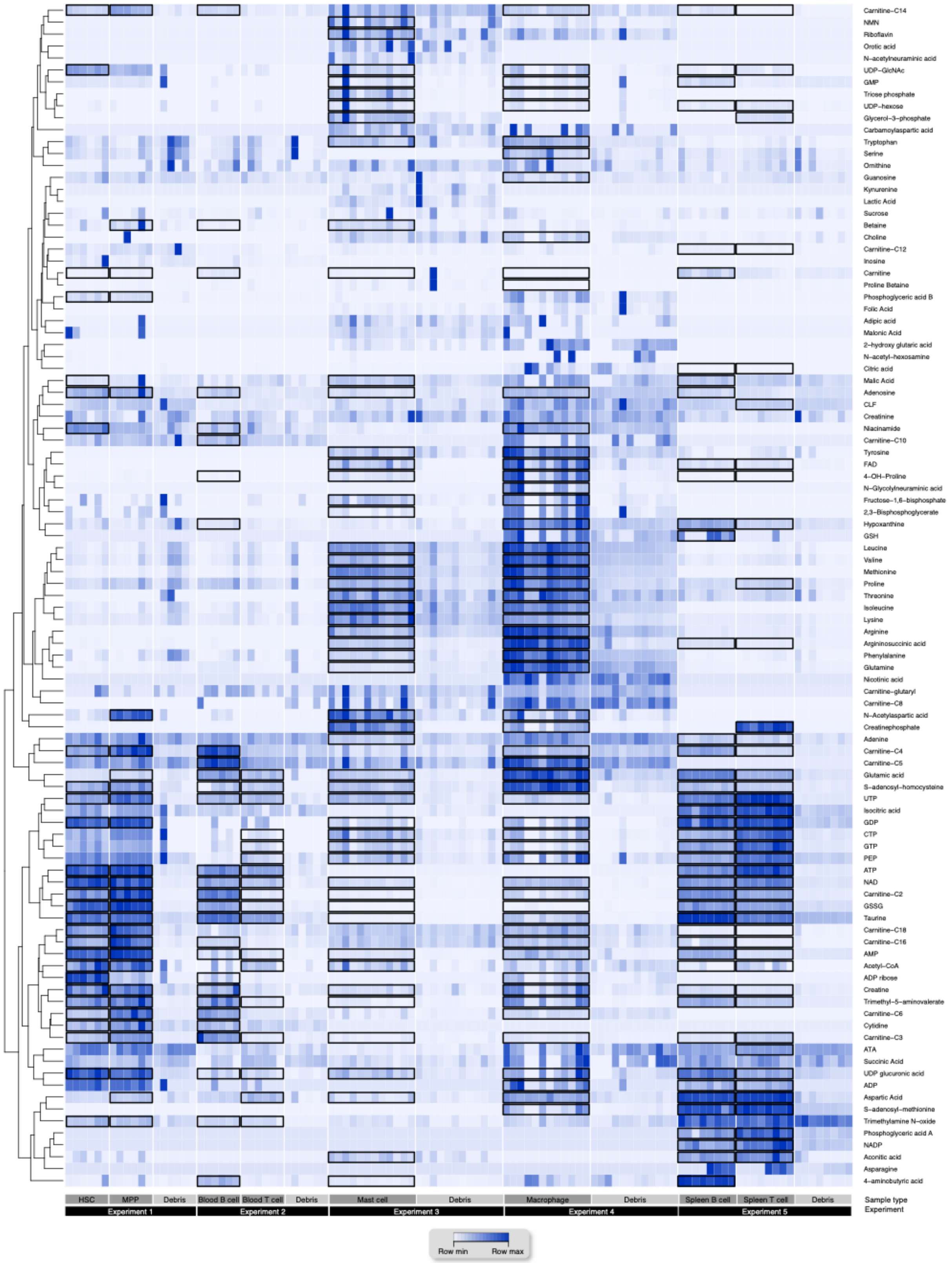

Supplementary Figure S 8: Heatmap of metabolite profiles of 8 different cell types (5000 events per replicate) obtained from 5 independent experiments. Black boxes indicate metabolites for which the signal intensity in cell extracts is significantly larger than in debris samples from the same experiment (FDR < 0.05 determined by one-sided Wilcoxon rank sum test corrected for multiple testing with the Benjamini & Hochberg method). The number of replicates is 6 in experiments 1 and 2, 12 in experiments 3 and 4, and 8 in experiment 5.
